# The protective effects of dietary Clostridium butyricum supplementation on hepatic ischemia reperfusion injury in rats

**DOI:** 10.1590/acb370904

**Published:** 2022-12-12

**Authors:** Xuan Yang, Hui Yu, Jingli Wei, Qiuyan Wei, Hui Huang, Jing Chen, Jianzhe Li, Shuyi Yu

**Affiliations:** 1 Undergraduate student. School of Clinical Medicine – Central South University – Changsha, China.; 2 MSc. Guangxi University of Chinese Medicine – The Affiliated Ruikang Hospital – Nanning, China.; 3 PhD. Guangxi University of Chinese Medicine – The Affiliated Ruikang Hospital – Nanning, China.; 4 PhD. Central South University – Advanced Research Centre – Changsha, China.

**Keywords:** Clostridium butyricum, Reperfusion Injury, Fatty Acids, Volatile, Inflammation, Gut Microbiota, Oxidative Stress

## Abstract

**Purpose:**

This study investigated the effects of oral administration of *Clostridium butyricum* (*C. butyricum*) on inflammation, oxidative stress, and gut flora in rats with hepatic ischemia reperfusion injury (HIRI).

**Methods:**

The rats from *C. butyricum* group were given *C. butyricum* for 5 days. Then, hepatic ischemia for 30 min and reperfusion for 6 h were performed in all the rats. After the animals were sacrificed, alanine transaminase (ALT), aspartate aminotransferase (AST), lipopolysaccharide (LPS) in serum, short-chain fatty acids (SCFAs), and gut microbiota composition in feces, and malondialdehyde (MDA), glutathione (GSH), tumor necrosis factor-α (TNF-α), interleukin 6 (IL-6), Toll-like receptor 4 (TLR4), nuclear factor-kappa Bp65 (NF-κBp65) and histological analysis in the liver were performed.

**Results:**

The rats given *C. butyricum* showed decreased ALT, AST, LPS, and MDA; improved GSH and histological damage; changes in SCFAs; declined TNF-α, IL-6, TLR4, and pNF-κBp65/NF-κBp65; and changes in the gut microbial composition, which decreased the Firmicutes/Bacteroidetes ratio and increased the relative abundance (RA) of probiotics.

**Conclusions:**

*C. butyricum* supplementation protected against HIRI by regulating gut microbial composition, which contributed to the decreased LPS and attenuation of inflammation and oxidative stress. These indicate *C. butyricum* may be a potent clinical preoperative dietary supplement for HIRI.

## Introduction

Liver ischemia reperfusion injury (I/R) is one of the primary causes of organ dysfunction associated with hepatectomy, liver transplantation, and shock syndrome^1^. Hepatic ischemia reperfusion injury (HIRI) is recognized as a two-stage process of ischemia-induced cell damage and reperfusion-induced inflammatory response, and is characterized by the activation of neutrophils, which triggers various pathological states, such as oxidative stress, inflammation, and apoptosis^1^^–^ 3. Many therapeutic studies have focused on attenuating or preventing HIRI; however, none have been completely successful.

Previous research has confirmed that TLR4/NF-κB signaling plays a vital role in the pathogenesis of HIR^4^^–^ 7. Intestinal microbial diversity and population are disturbed in a variety of liver diseases as well as in HIRI^8^^–^ 11, accompanied by impaired gut-barrier function, increased translocation of bacteria, and lipopolysaccharide (LPS) from the main components of the outer membrane of Gram-negative bacteria to the portal circulation. The inflammatory cascade is future promoting via LPS/TLR4 signaling in hepatocyte or Kupffer cells and induces the secretion of proinflammatory cytokines, such as tumor necrosis factor-α (TNF-α) and/or IL-6, after reperfusion and increased liver injury^10^^–^ 14. The effects of therapeutic approaches on gut microbiota during HIRI were mainly studied by using antibiotics, removing bacteria, blocking TLR4 signaling, or considering remote ischemic preconditioning^14^^–^ 16. To date, very few studies have aimed to ameliorate damage that occurs during HIRI through probiotics.

*Clostridium butyricum*—a Gram-positive, anaerobic, spore-forming bacillus—can consume undigested dietary fibers and produce short-chain fatty acids (SCFAs), specifically butyrate and acetate. The administration of *C. butyricum*, both in clinical practice and animal models, have had a beneficial effect on inflammatory bowel disease, intestinal tumor development, as well as cerebral ischemia, and obesity, without serious side effects in Asia^17^^,^ 18. The protection of *C. butyricum* on liver diseases has also been demonstrated well in animal models, such as the role of modulating TLR signaling pathways in high-fat, diet-induced liver injury^18^^–^ 19, and in relieving the CCl_4_-induced oxidative stress and inflammation in the liver by regulating NF-κB, IL-10 and the gut microbiota in mice^20^. It was firmly established that intravenous administration of butyrate protects LPS-induced liver injury by inhibiting the inflammation^21^^,^ 22. Partially building on this knowledge and inspired by research on the role of *C. butyricum* dietary supplementation in the disease pathophysiology of the liver and other organs, this study investigated the possibility that *C. butyricum* dietary supplementation attenuates HIRI in rats.

## Methods

### Culture of C. butyricum

*C. butyricum* (ATCC 19398, Baio Bowei Biotechnology Co., Ltd., Beijing, China) was cultured anaerobically at 37 °C for 24 h in tryptone soybean agar medium containing 5% defibrinated goat blood, 15 g tryptone, 5 g soybean peptone, 5 g NaCl, 15 g agarophyte, and 1.0 L distilled water. Bacteria were harvested, washed three times with phosphate buffer solution, then suspended in saline to obtain a bacterial suspension.

### Rat hepatic ischemia–reperfusion model

The animal experimental protocol was performed in compliance with the Chinese National Laboratory Animal-Guideline for Ethical Review of Animal Welfare and approved by the Institutional Animal Care and Use Committee of Central South University (No. 067016). Wistar rats were purchased from the Animal Resource Centre (Shanghai, China) and randomly assigned to one of the *C. butyricum*, I/R, or sham groups, with at least 10 rats in each group. The rats in the *C. butyricum* group were gavaged with 0.5 mL of saline containing 6.3 × 10^8^ CFU *C. butyricum*, once a day for 5 days^17^. The rats in the control and sham groups were given 0.5 mL of saline alone. As previously described, rats underwent 70% warm HIRI^5^^–^ 7. Briefly, a midline laparotomy was performed to expose the liver. The rats were anesthetized with pentobarbital sodium (50 mg/kg) (Shanghai Chemical Reagent Co., Ltd., Shanghai, China) by intraperitoneal injection. The blood supply to the left and median lobes was occluded in order to induce ischemia for 30 min. Then reperfusion for 6 h by removed the clamp. This procedure occurred on the I/R group rats. The rats from the sham group only received a switching abdominal surgery. Soon after surgery, the liver samples were kept on liquid nitrogen. The blood samples were centrifuged at 3000 r/min for 10 min, and the resultant plasma samples were stored at −80 °C for subsequent tests.

### Hematoxylin and eosin (H&E) staining (histological analysis)

Liver tissues were fixed in 10% buffered formalin and embedded in paraffin. Liver tissues with 5 μm thickness were then stained with H&E via standard procedures. The expert histologists evaluated the histopathological damage according to Suzuki’s criteria^23^ under a photomicroscope (Olympus BX51, Tokyo, Japan) in a blinded manner. The score ranged from 0 to 12, depending on the degree of cellular vacuolization (score: 0–4), inflammatory cell infiltration (score: 0–4), and necrosis (score: 0–4).

### Measurement of serum biochemical indicators and inflammation cytokines in hepatic tissue

Alanine transaminase (ALT) and aspartate aminotransferase (AST) were detected using a BS-830 chemistry system analyzer (Mindray Bio-medical Electronics Co. Ltd., Shenzhen, China) according to the manufacturer’s instructions of an available photometric assay kit (Xiamen Bioendo Technology Co., Ltd., Xiamen, China). The concentration of LPS in serum, IL-6, and TNF-α in hepatic tissue were measured using a commercially available ELISA kit (Shanghai Enzyme Link Biotechnology Co., Ltd, Shanghai, China).

### Analysis of malondialdehyde (MDA) and glutathione (GSH) levels in hepatic tissue

Hepatic tissues were homogenized, and centrifuged at 3,000 rpm for 15 min. The MDA levels in the supernatant were determined spectrophotometrically by thiobarbituric acid reactive substances, as described previously^23^. The absorbance was read at a 535 nm and the results were expressed as nmol MDA/g tissue.

The GSH measurements were performed using the Ellman procedure^24^, the absorbance at 412 nm was read, the extinction coefficient of 1.36 × 10^4^ mol L^−1^ cm^−1^ was used to calculate the GSH levels, and the results were expressed in μmol GSH/g tissue.

### Quantitative polymerase chain reaction (qPCR) assay

Total RNA was extracted from the liver tissue sample using TRIZOL Reagent (Invitrogen, Los Angeles, USA), the Nanodrop 2000 (Thermo Scientific, Waltham, USA), and gel electrophoresis was used to quantify and check the RNA quality. The cDNA was reverse transcribed from 2 μg RNA using the Transcript First Strand cDNA Synthesis Kit (Thermo Scientific, Waltham, USA). The PCR amplification products were quantified by GoTaq qPCR Master Mix (Promega, Madison, USA), following the appropriate procedure (95 °C for 10 s, 60 °C for 10 s, and 72 °C for 20 s for 45 cycles). The mRNA expression levels of the target genes were normalized to glyceraldehyde-3-phosphate dehydrogenase (GADPH). The primer pairs used in the experiment are shown in Table 1.

**Table 1:** Primer sequences of target genes for real-time PCR in the study.

Gene*	Forward	Reverse
GAPDH	GCGACTTCAACAGCAACTCCC	CACCCTGTTGCTGTAGCCGTA
TLR4	GGCTTCTAACCTCAACGCCT	ATGATTCTTGCCTGAGTTGCTT
NF-κBp65	CACCAAAGACCCACCTCACCG	CTTGCTCCAGGTCTCGCTTC
TNF-α	CCCCTCTATTTATAATTGCACCT	CTGGTAGTTTAGCTCCGTTT
IL-6	TCACTATGAGGTCTACTCGG	CATATTGCCAGTTCTTCGTA

* GADPH, Glyceraldehyde-3-phosphate dehydrogenase; TLR4, toll-like receptor 4; NF-κBp65, nuclear factor-kappa B; TNF-α, tumor necrosis factor α; IL-6, interleukin 6.

### Western blot analysis and antibodies

Hepatic tissues were crushed in a radioimmunoprecipitation assay lysis buffer with phosphatase and protease inhibitors (Roche, Mannheim, Germany) and quantified using a Pierce BCA Protein Assay Kit (Thermo Scientific, Waltham, MA, USA). The total protein samples were loaded, separated on SDS-PAGE gels, and then transferred to PVDF membranes (Merck Millipore, Darmstadt, Germany). The membranes were blocked with 5% skim milk and incubated overnight at 4 °C with NF-κBp65 (diluted 1:1,000), phospho-NFκBp65 (p-NF-κBp65, diluted 1:1,000), TLR4 (diluted 1:1,000), and GADPH (diluted 1:1,000) as primary antibodies, followed by incubation for 1 h at room temperature with peroxidase AffiniPure goat anti-rat-IgG (H+L; diluted 1:2,000) as the secondary antibody, which was purchased from Cell Signaling Technology (Beverly, MA, USA). Signals were imaged using enhanced chemiluminescence reagents (Bio-Rad, Hercules, CA, USA) and photographed with a ChemiDoc MP Imaging System (Bio-Rad).

### Evaluation of SCFAs in feces on GC-MS

To fecal samples weighing 50 mg, 0.5 mL of acetonitrile, and 10 μL of 1.0 mol/L HCl were added in a 1.5-mL centrifuge tube, stirred, immersed for 5 min in an ultrasonic bath for extraction, and then centrifuged for 10 min at 12,000 rpm. The solvent layer was analyzed using GC-MS (Ultra 2010 GCMS, Shimadzu). The chromatographic column was DB-WAX (0.25 mm × 0.25 μm × 30 m), the initial temperature was 40 °C for 1 min, and the rate of 10 °C/min was increased to 150 °C, further increasing it to 220 °C at 15 °C/min. The helium carrier gas flow rate was 1 mL/min. Standard SCFAs were purchased from Sigma-Aldrich (St. Louis, MO, USA). Quantitative analysis of the SCFAs in each sample was performed using the standard curve.

### Detection of the community diversity of gut flora using 16S rRNA sequencing

#### DNA extraction and PCR amplification

Fecal samples for rRNA sequencing were collected from the cecum of three randomly selected mice in each group. Microbial DNA in the fecal samples was extracted using the PowerSoil DNA Isolation Kit (MOBIO, Jefferson, MO, USA), in accordance with the manufacturer’s instructions. The V3+V4 region of bacterial 16S rRNA genes was amplified by PCR (98 °C for 2 min; 30 cycles at 98 °C for 30 s, 50 °C for 30 s, 72 °C for 60 s, and a final extension at 72 °C for 5 min) using the primers 338F (5’-ACTCCTACGGGAGGCAGC-3’) and 806R (5’-GGACTACHVGGGTWTCTAAT-3’). All PCR reactions were performed in triplicate in a 30- μL reaction mixture containing 6 μL of DNTPs (2 mmol/L), 0.9 μL of each primer, 0.6 μL of KOD FX Neo, 15 μL of KOD FX Neo Buffer, and 6.6 μL of ddH_2_O.

#### Illumina Novaseq6000 PE250 sequencing

Amplicons were extracted from 1.8% agarose gels and purified using the OMEGA DNA Gel Extraction kit (OMEGA, Norcross, GA, USA). The amplicons were then quantified by electrophoresis, collected in equimolar quantities, and analyzed with Illumina Novaseq6000 PE250 using standard protocols.

#### Statistical analysis

The results were expressed as mean ± standard deviation (SD). Differences among the experimental groups were evaluated using ANOVA. The statistical analysis was performed using GraphPad Prism 5 (La Jolla, San Diego, CA, USA). Significance was defined as a p-value < 0.05. A principal component analysis (PCA) was performed on the GCMS data using MetaboAnalyst 5.0 (https://www.metaboanalyst.ca/). QIIME2 was used to plot rarefaction curves and measure alpha (Chao and Shannon indexes) and beta diversities. The Operational Taxonomic Units of each sample were used for unweighted UniFrac distance-metric analysis. Principal coordinate analysis (PCoA) was performed based on the matrix of distance. The differences in phyla and genus levels were determined using a Mann–Whitney test to evaluate the impact of the *C. butyricum* dietary supplement on microflora diversity.

## Results

### *C. butyricum dietary* supplementation decreased the serum levels of ALT, AST, and LPS

**Figure 1:**
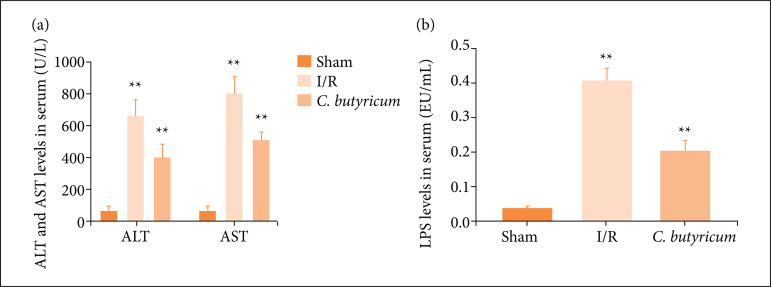
The **(a)** ALT, AST and **(b)** LPS levels in plasma. ^**^p < 0.01 vs. the sham group;

^##^p < 0.01 vs. the I/R group. Data are expressed as mean ± SD (n = 8 rats/group). LPS: lipopolysaccharide; ALT: alanine transaminase; AST: aspartate aminotransferase.

The serum levels of LPS and the acute liver damage/necrosis markers ALT and AST in the I/R group were remarkably increased compared with those in the sham group (p < 0.01). However, the ALT, AST, and LPS levels in the *C. butyricum* group were significantly reduced compared with those in the I/R group (p < 0.01), as shown in Fig. 1.

*C. butyricum* dietary supplementation attenuated HIRI-induced histopathology injury in the liver

**Figure 2:**
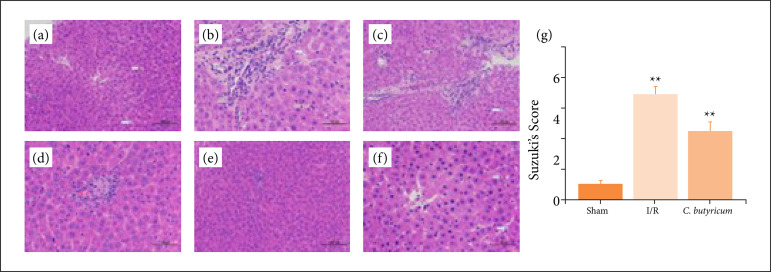
Histopathology photographs of hepatic tissue in the expe,rimental groups. Sham group: **(a)** 200× and **(b)** 400×, Liver parenchyma showed normal structure. I/R group: **(c)** 200× and **(d)** 400×. *C. butyricum* group: **(e)** 200× and **(f)** 400×. H&E stain, inflammatory infiltrate (black hollow arrow), cytoplasmic vacuolization (solid black arrow), liver necrosis (blue arrow). **(g)** Suzuki’s scores of hepatic tissue in the experimental groups.

^**^p < 0.01, vs. the sham group; ^##^p < 0.01 vs. the I/R group. Data are expressed as mean ± SD (n = 8 rats/group).

Regarding alterations in ALT and AST levels, hepatic injury was not observed in the sham group but was found in the I/R group, as evidenced by vacuole degeneration, inflammatory cell infiltration, hepatocyte necrosis, and higher Suzuki scores (Fig. 2). Furthermore, the rats in the *C. butyricum* group showed distinct decreases in hepatic injury and Suzuki scores (Fig. 2g).

### *C. butyricum* dietary supplementation attenuated HIRI-induced increases in MDA and GSH levels in the liver

**Figure 3:**
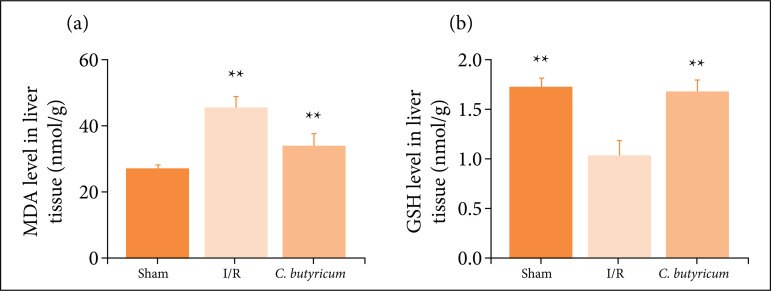
The **(a)** MDA and **(b)** GSH levels in the liver tissue. ^**^p < 0.01 vs. thesham group; ^##^p < 0.01 vs. the I/R group.

The GSH level was significantly decreased in the I/R group (p < 0.01) compared with the sham group. By contrast, the GSH level was significantly increased in the *C. butyricum* group compared with the I/R group (p < 0.01; Fig. 3a). The MDA level was increased in the I/R group compared with the sham group (p < 0.01), whereas in the *C. butyricum* group, it was decreased as compared with that in the I/R group (p < 0.01; Fig. 3b).

Data are expressed as mean ± SD (n = 8 rats/group). GSH: glutathione; MDA: malondialdehyde.

### TNF-α and IL-6 expression levels in mRNA and protein were attenuated in the *C. butyricum* group

**Figure 4:**
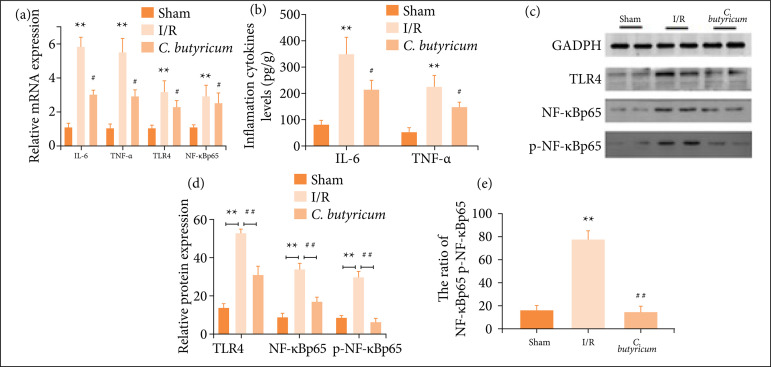
TNF-α, IL-6, TLR4 and NF-κBp-65 levels in **(a)** mRNA and **(b–e)** protein in the liver tissue.

^**^p < 0.01 vs. the sham group. ^#^p < 0.05, ^##^p < 0.01 vs. the I/R group. Data are expressed as mean ± SD (n = 8 rats/group).

Consistent with the hepatoprotective effects of the *C. butyricum* dietary supplement on HIRI observed in both the histological evaluation and biochemistry (plasma ALT/AST levels), the TNF-α and IL-6 levels in mRNA and protein were increased significantly in the I/R group compared with the sham group (p < 0.01) but were significantly reduced in the *C. butyricum* group compared with the I/R group (p < 0.05), as shown in Fig. 4A and B.

*The mRNA and protein expression levels of TLR4 and NF-*κ*Bp65 were decreased in the* C. butyricum *group*

The mRNA and protein expression levels of TLR4 and NF-κBp65 were higher in the I/R group than in the sham group (p < 0.01) but were decreased in the *C. butyricum* group compared with the I/R group (p < 0.05). Compared with those in the sham group, the p-NF-κB-p65 expression level and p-NF-κB-p65/NF-κBp65 ratio were significantly improved in the I/R group. In the *C. butyricum* group, the p-NF-κB-p65 expression level and p-NF-κB-p65/NF-κBp65 ratio were decreased as compared with those in the I/R group (p < 0.01), as shown in Fig. 4.

### The *C. butyricum* group and I/R or Sham group showed distinct SCFA profiles

**Figure 5:**
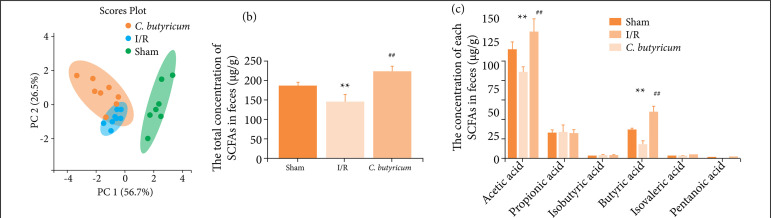
The SCFAs’ profiles in the hepatic tissue from the experimental groups’ feces. **(a)** PCA of SCFAs; **(b)** The total concentration of SCFAs in feces; **(c)** The concentrations of each SCFA in feces.

First, PCA was performed to evaluate the data, visualize the dominant patterns, and identify outliers in the populations. The SCFA profiles that contributed to the separation of the model was shown in Fig. 5a. The SCFA content (μg/g) in the fecal samples was strikingly different between the sham and I/R groups. The total amount of SCFAs was markedly lower in the I/R group than in the sham or *C. butyricum* group (Fig. 5b). In detail, compared with the sham or *C. butyricum* group, the I/R group showed lower acetic and butyric acid contents (Fig. 5c).

**p < 0.01, vs. the sham group. ^##^p < 0.01 vs. the I/R group. Data are expressed as mean ± SD (n = 8 rats/group).

### MiSeq sequencing results, Chao and Shannon indexes, and PCoA results

**Figure 6:**
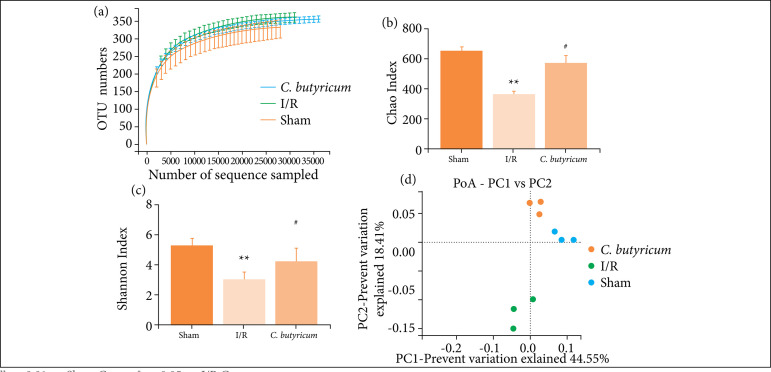
**(a)** The dilution curves of different samples; **(b)** the effects of *C. butyricum* dietary supplement on the Chao index; **(c)** Shannon index; and **(d)** PCoA plot, of fecal microflora after reperfusion for 6 h.

^**^p < 0.01 vs. Sham Group; ^#^p < 0.05 vs. I/R Group.

A total of 912,459 pairs of reads were obtained from the sequencing of nine samples. The dilution curve of each sample tended to be flat when the sequencing amount reached 20,000 (Fig. 6a), indicating that the sequencing depth reflected the flora. The Chao and Shannon indexes were significantly lower in the I/R Group (p < 0.05) than in the I/R group and were significantly higher in the *C. butyricum* group (p < 0.05; Fig. 6b,c). The PCoA results showed significant differences in the microflora structure of the feces from the distal ileum between the sham, I/R, and *C. butyricum* groups (Fig. 6d).

### Changes in microbial composition

**Figure 7:**
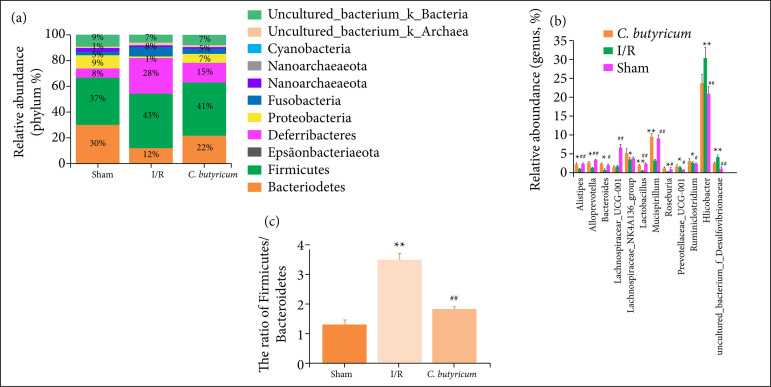
The effects of *C. butyricum* dietary supplement on the microbial composition in **(a)** the feces at the phylum, **(b)** genus levels, and the ratio of **(c)** Firmicutes/Bacteroidetes after reperfusion for 6h.

^**^p < 0.01 vs. the sham group; ^##^p < 0.01 vs. the I/R group. Data are expressed as mean ± SD (n =3 rats/group).

The microbial composition was quite different between the sham, I/R, and *C. butyricum* groups (Fig. 7). At the phylum level, the microflora community in the distal ileum in all three groups was dominated by Firmicutes, Bacteroidetes, Epsilonbacteraeota, Deferribacteres, and Proteobacteria. Compared with the sham group, the I/R group showed decreased relative abundance (RA) of Bacteroidetes and Deferribacteres and increased RA of Epsilonbacteraeota, Proteobacteria, and Firmicutes (p < 0.01). However, compared with I/R group, the *C. butyricum* group had significantly increased RA of Bacteroidetes and Deferribacteres and decreased RA of Epsilonbacteraeota and Proteobacteri (p < 0.01). In addition, compared with the I/R group, the RA of Firmicutes was not significantly different and the Firmicutes-to-Bacteroidetes ratio at the phylum level was decreased in the *C. butyricum* group (Fig. 7b).

The effect of *C. butyricum* supplementation on the RA of intestinal microflora at the genus level was also examined (Fig. 7c). The numbers of *Bacteroides*, *Alistipes*, Prevotellaceae_UCG-001, Lachnospiraceae_NK4A136_group, *Alloprevotella*, *Lactobacillus*, *Roseburia*, *Ruminiclostridium*, and *Mucispirillum* were significantly lower in the I/R group than in the sham group (p < 0.01). However, the *C. butyricum* group showed significantly increased numbers of these probiotics and Lachnospiraceae_UCG-001 except Prevotellaceae_UCG-001 and Lachnospiraceae_NK4A136_group compared with the I/R group. In addition, the numbers of Helicobacter and uncultured_bacterium_f_Desulfovibrionaceae were significantly greater in the I/R group than in the sham group. By contrast, treatment with *C. butyricum* significantly decreased the numbers of the last two pathogenic species (p < 0.01).

## Discussion

The present study established a rat model of HIRI and determined the serum ALT and AST levels and liver histology in rats in sham, I/R, and *C. butyricum* groups at 6 h after HIRI surgery. The elevated serum ALT and AST levels and obvious histological damage in the liver indicated that HIRI was induced successfully in the rat model. Oxidative stress plays a core role in the pathological mechanism of HIRI. MDA level is an indicator of lipid peroxidation, and GSH is a major endogenous antioxidant that plays an important role in antioxidant stress^1^^–^ 3. In this study, *C. butyricum* supplementation showed protective effects against HIRI, which was verified by the decreased serum ALT and AST levels and resolution of liver histological damage. This was related to decreased oxidative stress, indicating reduced MDA and restored GSH levels in the liver.

HIRI results in increased intestinal permeability and subsequent translocation of bacteria and LPS from the gut into circulation, following the induction of inflammatory signaling in the liver^11^^,^ 14. Previous researches have shown that *C. butyricum* contributes to the attenuation of inflammatory activation by inhibiting TLR signaling pathways in intestinal inflammatory diseases^17^^–^ 22 or decreasing LPS serum levels by maintaining the stability of the intestinal barrier in severe acute pancreatitis^23^. In the present study, the LPS serum concentration was increased significantly in the I/R group, and HIRI triggered metabolic endotoxemia. On the contrary, the LPS level in the *C. butyricum* group was decreased remarkably compared with that in the I/R group, which positively correlated with the histological damage and AST and ALT levels. It is reasonable that oral administration of *C. butyricum* can alleviate HIRI-induced endotoxemia.

LPS-TLR4 signaling can result in phosphorylation of the transcription factor NF-κBp65 and then regulate TNF-α and IL-6 levels. Several other inflammatory factors and the TLR4-NF-κBp65 signaling pathway are also vital in the pathogenesis of HIRI, consistent with previous researches^1^^–^ 3 ^,^ 9 ^–^ 13. The present study shows that oral administration of *C. butyricum* could inhibit the expressions of the inflammatory cytokines IL-6 and TNF-α, together with the downregulated TLR4 and NF-κBp65 expressions in rats with HIRI. Meanwhile, decreased pNF-κBp65 level and pNF-κBp65/NF-κBp65 ratio were observed. The results might be explained by the protective effects of the *C. butyricum* dietary supplement on HIRI in rats, mediated partially by the inhibition of the activation of LPS-TLR4-NF-κBp65 signaling-associated inflammation.

SCFAs generated by microbial organisms consuming indigestible fiber in the colon act as critical modulators of intestinal immune homeostasis, improving gastrointestinal barrier function and alleviating inflammation^15^^–^ 17. In general, the alteration of the fecal SCFA profile is due to gut microbial dysbiosis. In this study, the total content of SCFAs in the fecal samples was reduced under HIRI conditions. In particular, the acetic and butyric acid contents decreased significantly. On the contrary, oral administration of *C. butyricum* had the opposite effect. Previous research has confirmed that reductions in SCFA quantities and bacterial diversity in feces (e.g., for the loss of butyrogenic gut bacteria such as *F. prausnitzii*) are associated with changes in microbial composition in the gut of patients with various diseases^8^^–^ 17 and that *C. butyricum* has vital homeostatic functions and an inflammation inhibition effect in the human gut by improving butyric acid production^25^. This study is the first to report that oral administration of *C. butyricum* could reduce the amount of SCFAs and butyric acid level and restore liver function by reducing the acetic acid level in rats with HIRI.

HIRI induced dysbiosis of the intestinal microbiota in rats, as demonstrated by the MiSeq sequencing results. The Chao index is often used to estimate the total number of species in ecology, which positively correlates with the total number of species. The Shannon index positively correlates with community diversity in samples. In this study, these indexes demonstrated that HIRI decreased the abundance and diversity of the microflora community, which were significantly increased by oral treatment with *C. butyricum*.

HIRI-induced dysbiosis of the intestinal microbiota in rats has also been verified at the phylum and genus levels. *Bacteroides*, *Alistipes*, *Alloprevotella*, and *Lactobacillus* belong to Bacteroidetes, while *Roseburia*, Lachnospiraceae_UCG-001, and *Ruminiclostridium* belong to Firmicutes, which are probiotics involved in the production of SCFAs, such as butyrate, propionate, and acetate^26^^,^ 27. *Mucispirillum*, a member of the phylum Deferribacteres, impedes pathogenic bacteria by, for example, restricting the access of *C. difficile* to mucosal sugars and impairs pathogen colonization in antibiotic-treated mice^28^. Helicobacter and *Desulfovibrio* are harmful, Gram-negative bacteria belonging to Proteobacteria and Epsilonbacteraeota, and overgrowth of these pathogens can lead to elevated LPS levels^29^^,^ 30. The results showed that the levels of the six probiotics decreased and that the growth of the two pathogens increased during HIRI but recovered after *C. butyricum* treatment. Although no significant difference in Firmicutes abundance was observed between the I/R and *C. butyricum* groups, the Firmicutes-to-Bacteroidetes ratio decreased at the phylum level and *Roseburia* growth increased at the genus level, indicating that *C. butyricum* supplementation regulates the intestinal flora structure and changes the microbial composition. In addition, the effect of *C. butyricum* on Prevotellaceae_UCG-001 was not shown in the experiment. Prevotella hydrolases specialize in the decomposition of plant fibers. By contrast, *Bacteroides* was associated with diets rich in animal protein and saturated fatty acids. In general, the abundance of *Bacteroides* is inversely proportional to that of *Prevotella* 31. This study found that *C. butyricum* could restore the Bacteroidetes abundance reduced by HIRI but had no effect on reduced Prevotella growth. The previous findings confirmed that the consumption of *C. butyricum* benefited the gut microbial ecosystem, possibly by increasing the production of certain beneficial bacterial taxa such as *Lactobacillus* and *Bifidobacterium* and decreasing the growth of *Enterococcus* and/or Enterobacteriaceae^32^. In this study, we observed differences in the RA of *Lactobacillus* in the genus in the three groups and report for the first time that *C. butyricum* regulated the growth of *Alistipes*, *Roseburia*, and *Mucispirillum*.

## Conclusion

Therefore, during HIRI, the decreased amount of SCFAs is due to changes in intestinal flora. *C. butyricum* supplementation not only enhances the production of butyric acid in the gut but also reconstructs the intestinal microecology by regulating the growth of butyric or acetic acid-producing probiotics, inhibiting the abundance and colonization of pathogenic bacteria and thereby maintaining intestinal health, which contributes to reduced circulatory LPS level and the level of the LPS associated with the TLR4, NF-κBp65, TNF-α, and IL-6 signaling pathways. Although further studies are required to elucidate the precise mechanisms of the effects of *C. butyricum* on diverse combinations of genetic and environmental factors in the gut during HIRI, our findings suggest that *C. butyricum* may be a potential clinical nutritional supplement for the prevention of HIRI.
